# Lack of Exercise is Associated With Psychological Distress in Patients With Obstructive Sleep Apnea Syndrome

**DOI:** 10.7759/cureus.93074

**Published:** 2025-09-23

**Authors:** Hiroaki Kataoka, Nobuyuki Miyatake

**Affiliations:** 1 Department of Physical Therapy, Okayama Healthcare Professional University, Okayama, JPN; 2 Department of Hygiene, Faculty of Medicine, Kagawa University, Kagawa, JPN

**Keywords:** daytime sleepiness, exercise habit, exercise therapy, obstructive sleep apnea syndrome (osas), psychological distress, the kessler psychological distress scale (k6)

## Abstract

Background and objective

Obstructive sleep apnea syndrome (OSAS) is linked not only to cardiometabolic disorders but also to psychological distress such as depression and anxiety. Although continuous positive airway pressure (CPAP) and regular exercise have been shown to improve psychological well-being, the specific contribution of exercise habits to mental health in OSAS patients, particularly those receiving CPAP therapy, remains unclear. This study aimed to identify clinical and lifestyle-related factors associated with psychological distress in patients with OSAS, focusing particularly on exercise habits.

Methods

We conducted a cross-sectional analysis of 1,204 OSAS patients receiving CPAP therapy. Psychological distress was assessed using the six-item Kessler scale (K6), and a cutoff score of ≥5 was used to define the distress group. Daytime sleepiness was measured using the Japanese version of the Epworth Sleepiness Scale (JESS). Logistic regression was performed to identify predictors of psychological distress, with sex, age, BMI, apnea-hypopnea index (AHI), JESS score, and exercise habit included as independent variables.

Results

Among the participants, 153 (12.7%) were classified as experiencing psychological distress. Logistic regression revealed that higher JESS scores (odds ratio (OR) = 1.12, 95% confidence interval (CI): 1.08-1.16, *p* < 0.001) and absence of exercise habits (OR = 0.48, 95% CI: 0.32-0.72, *p* < 0.001) were significantly associated with psychological distress. Male sex was associated with lower odds of distress (OR = 0.61, 95% CI: 0.38-0.96, *p* = 0.032). No significant associations were observed for age, BMI, or AHI.

Conclusions

Lack of regular exercise and increased daytime sleepiness were independently associated with psychological distress in OSAS patients. These findings highlight the importance of exercise promotion and sleep management in addressing mental health in this population. Physical therapists may play a vital role in implementing lifestyle interventions to improve both physical and psychological well-being among individuals with OSAS.

## Introduction

Obstructive sleep apnea syndrome (OSAS) is a sleep-related breathing disorder characterized by recurrent episodes of apnea and hypopnea during sleep. Clinically, it often presents with loud snoring, witnessed apneas, nocturnal awakenings, and excessive daytime sleepiness, which may lead to impaired concentration and fatigue. In addition to these characteristic symptoms, OSAS is associated with an increased risk of cardiovascular disease, diabetes, and metabolic syndrome [1]. The prevalence of OSAS is increasing worldwide in parallel with aging populations and rising obesity rates, and it is estimated to affect a substantial proportion of middle-aged and older adults. In addition to its well-established physical health risks, OSAS has been increasingly recognized as a contributor to mental health problems, with accumulating evidence linking it to psychological distress, including depression, anxiety, and reduced quality of life [2]. These mental health burdens may adversely influence treatment adherence, occupational performance, and overall well-being.

In this context, psychological distress in the present study was assessed using the six-item Kessler scale (K6) [3]. Several studies have examined the relationship between OSAS severity and mental health outcomes. However, most have focused on objective indicators such as the apnea-hypopnea index (AHI), and less attention has been paid to modifiable lifestyle factors. Among such factors, regular physical activity is known to improve mood, reduce stress, and enhance sleep quality, and these factors may collectively contribute to improved psychological well-being [4]. Previous interventional studies have also demonstrated that continuous positive airway pressure (CPAP) therapy and lifestyle modifications such as regular physical activity can improve mood, anxiety, and psychological distress in patients with OSAS [5]. These findings suggest that psychological distress in this population is not necessarily irreversible but may improve with appropriate treatment and behavioral interventions. However, the specific contribution of exercise habits to psychological well-being in patients with OSAS, especially those receiving CPAP therapy, is not fully clear.

Physical therapists frequently observe that patients with regular exercise habits demonstrate greater emotional stability and higher engagement in daily life activities [6-8]. Based on these observations, we hypothesized that patients with OSAS who lack regular exercise habits may be more prone to psychological distress. Therefore, this study aimed to identify clinical and lifestyle-related risk factors for psychological distress in patients with OSAS, with a particular focus on the presence or absence of exercise habits.

## Materials and methods

Study design and participants

This cross-sectional study involved a sub-analysis of a previous study investigating the relationship of locomotive syndrome with the health-related quality of life in patients with OSAS [9]. This analysis included 1,204 patients with OSAS (1,039 males and 165 females; mean age: 61.4 ± 12.9 years) who were receiving CPAP therapy at KKR Takamatsu Hospital (Kagawa, Japan) during November-December 2016. Patients with acute or chronic musculoskeletal disorders, other severe neurological or endocrine disorders, or a history of stroke were excluded. All participants provided written informed consent, and the study protocol was approved by the Research Ethics Committee (approval number: E111) of KKR Takamatsu Hospital and complied with the principles of the Declaration of Helsinki.

Evaluation of clinical parameters

This study collected the following clinical data: sex, age, height, weight, BMI, systolic blood pressure, and diastolic blood pressure. Additionally, information regarding the treatment period of CPAP therapy and AHI was collected from the clinical records. The severity of OSAS was calculated based on the patient’s AHI (number of apneas and hypopneas per hour) and classified into three categories: mild (5-15/h accompanied by typical clinical symptoms), moderate (16-29/h), and severe (≥30/h) [10]. Trained medical staff conducted interviews and performed assessments using the Japanese version of the Epworth Sleepiness Scale (JESS) [11]. The JESS questionnaire is designed to evaluate daytime sleepiness and comprises eight questions related to daily activities. Participants were asked to evaluate the likelihood of dozing off or falling asleep in specific situations, with responses scored as 0 points (no likelihood of dozing off), 1 point (some likelihood of dozing off), 2 points (moderate likelihood of dozing off), or 3 points (high likelihood of dozing off). A total score exceeding 10 points indicates excessive daytime sleepiness. Additionally, they collected information regarding patients’ exercise habits (aerobic exercise [e.g., walking or jogging] for >30 min three times per week within six months) [12].

Psychological distress was assessed using the K6, which is a self-administered questionnaire for assessing depressive symptoms and anxiety within the previous four weeks [3]. Each item is scored on a 5-point scale (0-4), with the total score ranging from 0 to 24. Higher scores indicate greater psychological distress. The K6 has been widely used to screen for psychological distress and validated in both general and clinical populations [13]. Based on previous studies [13], a cutoff score of 5 was used to classify participants into the non-psychological distress group (NPD; K6 score: 0-4) or the psychological distress group (PD; K6 score: 5-24).

Statistical analysis

Data are expressed as mean ± standard deviation (SD). The Shapiro-Wilk test was used to assess the distribution normality of continuous variables. Based on the distribution, between-group comparisons were performed using the unpaired t-test or Mann-Whitney U test. Categorical variables were compared using the χ² test. Logistic regression analysis was conducted to identify risk factors for psychological distress. The dependent variable was the presence (1) or absence (0) of psychological distress. Independent variables included sex (female = 0, male = 1), age, BMI, AHI, JESS score, and exercise habits (absence = 0, presence = 1). Statistical significance was set at *p* < 0.05. All statistical analyses were performed using SPSS Statistics version 29.0.1 (IBM Corp., Armonk, NY).

## Results

Table 1 provides the clinical characteristics of participants in each group. Compared to the NPD group (n = 1,051), the PD group (n = 153) had a significantly lower proportion of males and a significantly lower proportion of patients with regular exercise habits. The PD group participants were also significantly younger in age, had a significantly higher JESS score, and a higher degree of daytime sleepiness.

**Table 1:** Comparison of clinical parameters between patients with and without psychological distress ^*^Residual AHI during CPAP therapy SD: standard deviation; BMI: body mass index; SBP: systolic blood pressure; DBP: diastolic blood pressure; CPAP: continuous positive airway pressure; AHI: apnea hypopnea index; JESS: Japanese version of the Epworth Sleepiness Scale; PD: psychological distress; NPD: non-psychological distress

	PD group	NPD group	Test statistic			P-value
			U	Z	χ^2^	
N	153	1,051	-	-	-	
Sex, male, n (%)	124 (81.0)	915 (87.1)	-	-	4.085	0.043
Age, years, mean ± SD	58.6 ± 14.7	61.7 ± 12.6	70220.0	-2.534	-	0.011
Height, cm, mean ± SD	165.5 ± 9.9	165.4 ± 8.1	82644.5	0.558	-	0.577
Body weight, kg, mean ± SD	78.2 ± 18.3	75.6 ± 14.6	87732.0	1.824	-	0.068
BMI, kg/m^2^,mean ± SD	28.5 ± 6.2	27.5 ± 4.4	83554.0	0.785	-	0.433
SBP, mmHg, mean ± SD	135.0 ± 11.6	134.1 ± 11.7	84704.0	1.073	-	0.283
DBP, mmHg, mean ± SD	77.0 ± 10.2	75.7 ± 10.0	87106.5	1.673	-	0.094
Treatment period of CPAP therapy, months, mean ± SD	68.7 ± 52.9	69.9 ± 49.6	78146.5	-0.561	-	0.575
AHI^*^, mean ± SD	3.21 ± 3.63	2.79 ± 3.31	83389.0	0.744	-	0.457
Normal (n/%)	123 (80.4)	923 (87.8)	-	-	7.150	0.067
Mild, n (%)	27 (17.6)	118 (11.2)				
Moderate, n (%)	3 (2.0)	8 (0.8)				
Severe, n (%)	0	2 (0.2)				
JESS score, mean ± SD	8.10 ± 5.43	5.49 ± 4.13	104236.5	5.952		< 0.001
Exercise habit, n (%)	36 (23.5)	421 (40.1)	-	-	15.49	< 0.001

There were no significant differences between groups in terms of BMI, AHI, or treatment period for CPAP therapy. In logistic regression analysis (Table 2), the absence of exercise habits and high JESS scores were independently associated with an increased risk of psychological distress, even after adjusting for sex, age, BMI, and AHI. The odds ratio (OR) for lack of exercise habits (OR = 0.48, 95% confidence interval (CI): 1.08-1.16, *p* < 0.001) suggested a moderate but clinically significant reduction in the likelihood of belonging to the NPD group. Conversely, the JESS scores (OR = 1.12, 95% CI: 1.08-1.16, *p* < 0.001) indicated that increased daytime sleepiness was significantly associated with the risk of psychological distress. Male sex was associated with a reduced risk of psychological distress (OR = 0.61, 95% CI: 0.38-0.96, *p* = 0.032). Age exhibited a significant difference in univariate analysis; however, this association weakened in the multivariate model, suggesting potential confounding by other factors, such as daytime sleepiness or exercise habits. Similarly, AHI did not exhibit a significant association with psychological distress in either univariate or multivariate analyses, suggesting that the severity of OSAS based on the frequency of respiratory events may be a less reliable predictor of psychological distress compared to subjective symptoms or lifestyle factors.

**Table 2:** Logistic regression analysis of the relationship of clinical parameters with psychological distress JESS: Japanese version of the Epworth Sleepiness Scale; OR: odds ratio; CI: confidence interval

Variables	Wald	OR (95% CI)	P-value
Sex			
Female		1.00	
Male	4.595	0.61 (0.38–0.96)	0.032
Exercise habit (yes)		1.00	
Exercise habit (no)	12.76	0.48 (0.32–0.72)	< 0.001
JESS	40.79	1.12 (1.08–1.16)	< 0.001

## Discussion

Our findings indicated a significant association of psychological distress with both lack of regular exercise and increased daytime sleepiness in patients with OSAS. Specifically, patients without habitual physical activity experienced an increased risk of psychological distress. These findings are consistent with those of previous reports indicating the mental health benefits of regular physical activity across diverse populations [14].

The observed link between elevated psychological distress and OSAS in our study likely reflects a multifaceted interaction of neurophysiological, inflammatory, and behavioral mechanisms. Chronic intermittent hypoxia and sleep fragmentation are hallmarks of OSAS that can lead to dysregulation of the hypothalamic-pituitary-adrenal (HPA) axis, resulting in hypercortisolemia and persistent stress activation that have been associated with depressive and anxious symptomatology [15]. In addition, sympathetic overactivity within the autonomic nervous system appears to be most prominently involved. OSA patients exhibit chronic daytime sympathetic activation, which has been shown to improve with effective CPAP therapy [16,17]. This sympathetic hyperactivity contributes to cardiovascular strain and heightened stress responses, thereby exacerbating psychological distress. Additionally, recurrent nocturnal hypoxia induces systemic inflammation, characterized by elevated cytokines such as interleukin‑6 and TNF‑α that have been implicated in the development of mood disturbances [18].

Neuroimaging studies further support these pathophysiological underpinnings. For example, patients with untreated moderate to severe OSAS possess reduced gray matter volumes in key mood-regulating brain regions, including the prefrontal cortex and hippocampus, and altered functional connectivity within networks mediating emotional regulation [19,20]. These structural and functional brain alterations are hypothesized to contribute to impaired emotional control, increased irritability, and decreased psychological well‑being. From a behavioral standpoint, excessive daytime sleepiness and fatigue impair daily functioning, social engagement, and the ability to maintain regular physical activity, thereby amplifying feelings of helplessness and social withdrawal [21]. Poor subjective sleep quality and reduced daytime alertness may also promote negative cognitive appraisals, maladaptive coping, and diminished resilience, all contributing to elevated psychological distress [21].

Exercise stimulates the release of mood-regulating neurotransmitters, including serotonin and dopamine. Further, it simultaneously reduces the activity of excitatory neurotransmitters such as glutamate that are linked to anxiety and depression [22]. By modulating inflammatory markers, altering neurotransmitter levels, and influencing brain structure and function, physical activity can contribute to improved mood, reduced anxiety and depressive symptoms, and improved psychological well-being. Additionally, regular physical activity enhances sleep architecture and mitigates the severity of OSAS symptoms, and this may further reduce psychological distress. Additionally, we observed that male patients exhibited a reduced risk of experiencing psychological distress. This sex-based difference may be attributed to psychological and sociocultural factors, including sex differences in stress perception, emotional expression, and help-seeking behaviors.

In our analysis, AHI was not significantly associated with psychological distress, a finding corroborated by prior studies indicating that objective indices of OSAS severity may not align with psychological outcomes [2,23]. While AHI quantifies the frequency of apneas/hypopneas, it does not account for the depth of oxygen desaturation, extent of sleep fragmentation, or other qualitative aspects of sleep disruption factors more closely linked to mood and cognitive impact [23]. Moreover, subjective disease burden, such as perceived fatigue, sleepiness, and reductions in quality of life, frequently demonstrates stronger correlations with mental health outcomes than does AHI itself. For example, one study reported that even patients with mild-to-moderate OSAS (AHI < 15) experienced significant psychological distress and lower health-related quality of life [24].

These discrepancies may reflect inter-individual variation in symptom sensitivity, sleep reactivity, and resilience profiles. Finally, potential confounders not measured in our study, such as socioeconomic stressors, comorbid chronic conditions (e.g., pain, metabolic disease), and psychological factors, may influence mental health independently of respiratory event frequency [25]. Collectively, these considerations suggest that comprehensive assessment using both physiological and subjective measures (e.g., K6, JESS) provides a more meaningful evaluation of psychological risk in OSAS patients than does reliance on AHI alone.

From a physical therapy perspective, these findings highlight the importance of incorporating exercise interventions into the routine care of patients with OSAS for improved physical and mental health. Screening for psychological distress using tools such as the K6, combined with evaluation of exercise habits and daytime sleepiness, could facilitate early identification of at-risk individuals. Multidisciplinary collaboration, including physical therapists, sleep specialists, and mental health professionals, may optimize both mental and physical outcomes.

This study has several limitations. First, given the cross-sectional design, we could not establish a causal relationship between exercise habits and psychological distress. Second, exercise habits were assessed through self-report interviews, and this may be prone to recall bias. Additionally, the accuracy of the physical activity assessment may be limited, as we did not perform objective measurements using accelerometers. Third, we did not consider potential confounders that may influence psychological distress, including socioeconomic status, substance use, or pre-existing mental health conditions. Future longitudinal and interventional studies are warranted to clarify causal pathways and confirm the effects of exercise interventions on mental health in patients with OSAS and determine strategies to enhance adherence to such interventions. Fourth, our study did not capture detailed CPAP parameters such as pressure settings or nightly usage hours. Although we confirmed that the mean treatment period of CPAP therapy was approximately 69 months, the lack of parameter data limits the interpretation of how treatment adherence or intensity might have influenced psychological outcomes. Prior studies indicate that average CPAP usage of about 4.5 hours per night can lead to improvements in quality of life and emotional functioning [26]. Therefore, future research should incorporate more comprehensive CPAP adherence metrics.

## Conclusions

Physical inactivity and increased daytime sleepiness were significantly associated with psychological distress in patients with OSAS. Promoting regular physical activity may serve as an effective strategy for improving mental health in this population. Physical therapists can play a crucial role in delivering exercise-based interventions and providing psychosocial support for individuals affected by OSAS.
